# 

**Published:** 1895-12-21

**Authors:** 


					? ?   AKSOLUTkLV FUkk, thereiore BEST. ihsumuusk *jst, lm*.
CADBURYS * ?* ** CADBURY'S
COCOA ^TH COCOA
I" The standard of highest purity at present ??> *? W?J NO ALKALIES USED,
attainable."?Lanect.
No. 482. Vol. XIX.
Saturday,
Deo. 21st, 1895.
WITH tXTRA NUKSINGiSUi'l'LtWlENT. ^^^.w^'postfree.
?wgg.?ga.^'ia"" ^assauaata

				

